# New Zwitterionic Polymer as a Highly Effective Salt- and Calcium-Resistant Fluid Loss Reducer in Water-Based Drilling Fluids

**DOI:** 10.3390/gels8110735

**Published:** 2022-11-11

**Authors:** Luman Liu, Jinsheng Sun, Ren Wang, Fan Liu, Shifeng Gao, Jie Yang, Han Ren, Yuanzhi Qu, Rongchao Cheng, Yuan Geng, Zhenbo Feng

**Affiliations:** 1 College of Petroleum and Nature Gas Engineering, Southwest Petroleum University, Chengdu 610500, China; 2 CNPC Engineering Technology R&D Company Ltd., Beijing 102206, China; 3 College of Chemistry and Chemical Engineering, Southwest Petroleum University, Chengdu 610500, China; 4 Daqing Drilling & Exploration Engineering Corporation No. 2 Drilling Co., Daqing 163413, China

**Keywords:** zwitterionic, gel, high-temperature resistance, salt- and calcium-resistant, fluid loss reducer

## Abstract

To control the filtration loss of drilling fluids in salt–gypsum formations, a novel type of zwitterionic polymer gel (DNDAP) was synthesized by free radical polymerization, which was used as a salt- and calcium-resistant fluid loss reducer for water-based drilling fluids (WBDF). DNDAP was prepared with N, N-dimethylacrylamide (DMAA), N-vinylpyrrolidone (NVP), Diallyl dimethyl ammonium chloride (DMDAAC), 2-acrylamide-2-methylpropaneonic acid (AMPS), and isopentenol polyether (TPEG) as raw materials. Fourier transform infrared spectroscopy (FT-IR) and proton nuclear magnetic resonance (^1^H-NMR) were used to characterize the composition and structure of the DNDAP copolymer. The thermal stability of DNDAP was evaluated by the use of thermogravimetric analysis (TGA). WBDF with DNDAP was analyzed for zeta potential and particle size and the corresponding filter cake underwent energy dispersive spectrum (EDS) analysis and scanning electron microscope (SEM) analysis. The results showed that the thermal decomposition of DNDAP mainly occurred above 303 °C. DNDAP exhibits excellent rheological and filtration properties in water-based drilling fluids, even under high-temperature aging (up to 200 °C) and high salinity (20 wt% NaCl or 5 wt% CaCl_2_) environments. The strong adsorption effect of DNDAP makes the particle size of bentonite reasonably distributed to form a dense mud cake that reduces filtration losses.

## 1. Introduction

With the rapid growth of oil demand and the decline of shallow oil and gas resources, deep oil and gas have become the focus of oil and gas exploration and development as a result of the increased oil demand [1,2]. The drilling fluid requirements in deep reservoirs are higher due to their high temperatures, pressures, and complex geological conditions (mainly the salt paste layer). It has been found that gypsum is a potentially reliable reservoir for oil and gas [3,4,5,6,7]. It is estimated that overburdened materials such as gypsum and salt rocks constitute nearly 30% of the overburden of large oil fields throughout the world [8,9,10]. The gypsum layer contains a large amount of Na^+^ and Ca^2+^. The drilling fluid is highly likely to be contaminated by high temperatures and cations during the drilling process [11,12]. Therefore, drilling fluids require high-temperature resistance and salt and calcium resistance in oil and natural gas drilling operations. Drilling fluids carry and suspend cuttings, stabilize wellbore, balance formation pressure, cool and lubricate drill tools, transfer energy, and assist in rock breaking during drilling. In the current drilling fluid market, both water-based and oil-based drilling fluids are commonly used for the drilling process. Even though oil-based drilling fluids are superior to water-based drilling fluids when it comes to stability at high temperatures and pressures, they are more expensive, make it difficult to dispose of cuttings, and pose a serious risk to the environment [13,14,15,16]. Considering the low cost and environmental friendliness of water-based drilling fluid, it has been a key research area. Essentially, water-based drilling fluids consist of bentonite, various polymeric agents (diluents, fluid loss reduction agents, shale inhibitors, etc.), and weighting materials. The fluid loss reducer has been widely studied as one of the most critical treatment agents in water-based drilling fluids. Currently, the commonly used fluid loss additives are generally divided into naturally modified materials and polymer fluid loss additives. The modified natural materials usually cannot withstand temperatures higher than 180 °C, or even 160 °C, and cannot meet the requirements of deep well drilling operations. Polymer materials are widely used in petrochemistry, environmental protection, medicine and biology, and other fields because of their high-temperature resistance, high strength, strong toughness, and other advantages. Therefore, polymers are synthesized as filter loss reduction agents in deep well drilling operations [17,18,19,20,21,22,23,24,25]. Li et al. [26] synthesized a polymer, SPL, by chemically cross-linking starch, polyphenols, and lignosulfonate in a filtration volume of 7.0 mL at 150 °C in water-based drilling fluid. In addition, SPL was resistant to 0.75 wt% CaCl_2_ and 7.5 wt% NaCl at 150 °C. Shan et al. [27] prepared nano-SiO_2_ graft copolymers by inverse emulsion polymerization. It was found that EAANS polymers were prepared using acrylamide (AM), AMPS, NVP, and KH570-modified nano-silica (M-SiO_2_). Even after aging at 150 °C, EAANS still exhibits good filtration loss and rheological properties when NaCl or CaCl_2_ concentrations are up to 36 or 30 wt%, respectively. Liu et al. [28] synthesized an amphoteric polymer, ADD, by using AMPS, AM, and DMDAAC, which acts as an anti-calcium pollution filtration additive. The API filtration volume was maintained at 9.6 mL after hot rolling at 150 °C in 11.1 wt% CaCl_2_-contaminated sodium bentonite-based mud.

In recent years, amphoteric polymers have received extensive attention due to their excellent hydration, adsorption, biocompatibility, and stability. Up to now, many new and functional zwitterionic polymers have been synthesized and applied in the petroleum industry, biomedical materials, drug synthesis, sewage treatment, and other fields [29,30]. In this study, a new zwitterionic copolymer gel, DNDAP, was prepared by using DMAA, NVP, DMDAAC, AMPS, and TPEG monomers as raw materials. Since DMAA is the backbone of the copolymer, it is more hydrolysis-resistant than the AM monomers commonly used in copolymers. NVP contains a five-membered ring structure with high steric hindrance, which can enhance backbone rigidity [31,32,33]. In addition, carbonyl groups in NVP can form hydrogen bonds with amide groups, thus inhibiting the decomposition of adjacent amide side groups. As a result of the strong hydration provided by AMPS anionic monomers, the sulfonic acid group is not sensitive to external cations or high temperatures, which is beneficial for improving the copolymer’s tolerance to temperature and salt [34,35,36]. A five-membered intramolecular ring bond is formed on the macromolecular chain of the synthesized copolymer as a result of the use of DMDAAC, which improves the rigidity and resistance of the copolymer at different temperatures and in different salt environments. The molecular structure of TPEG is comb-like, and the long polyethylene oxide chain in the comb-like structure can invade the dispersion medium and improve the stability of the polymer through steric hindrance [37,38]. In this paper, TPEG monomer was introduced into a zwitterionic copolymer for the first time, which is beneficial for improving the high-temperature resistance and salt- and calcium-resistance of the polymer, and provides a new idea for the subsequent study of filtration loss agents.

## 2. Results and Discussion

### 2.1. Characterization of DNDAP Copolymer 

**Figure 1:**
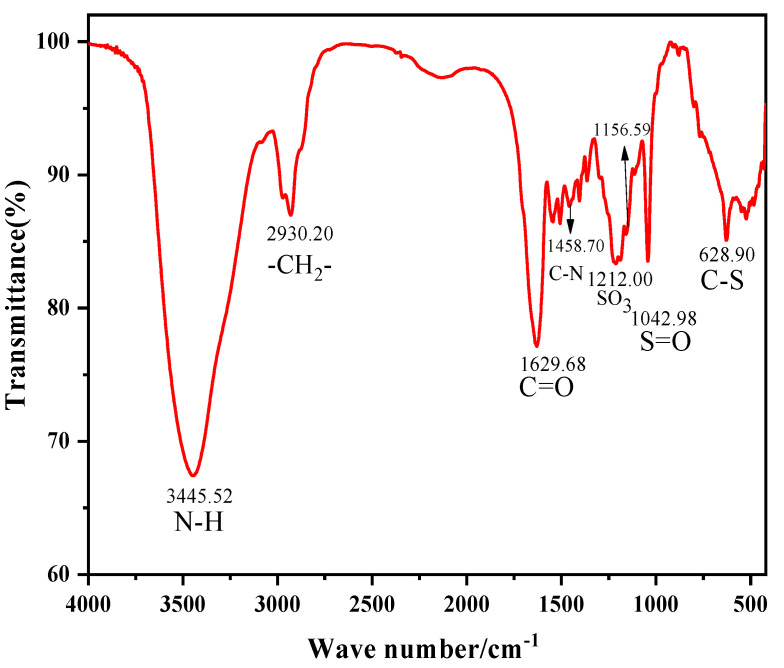
FTIR spectrum of DNDAP.

A FTIR spectrum of DNDAP is shown in Figure 1, with the absorption peak at 3445.52 cm^−1^ corresponding to the stretching vibration of the N–H bond in DMAA, AMPS, DMDAAC, and NVP. The molecular main chain stretching vibration of CH2 in DNDAP is responsible for an absorption peak at 2930.20 cm^−1^. It can be seen from the figure that there is an obvious absorption peak at 1629.68 cm^−1^, which corresponds to the tensile vibration of C=O in DMAA and NVP. C–N stretching was observed at approximately 1458.70 cm^−1^ in DMAA, DMDAAC, and NVP. It has been determined that SO_3_ from AMPS has a stretching vibration at 1212.00 cm^−1^ [39]. The absorption peak at 1156.59 cm^−1^ is attributed to TPEG. A C-S absorption band from AMPS was responsible for the characteristic peak at 628.90 cm^−1^.

**Figure 2:**
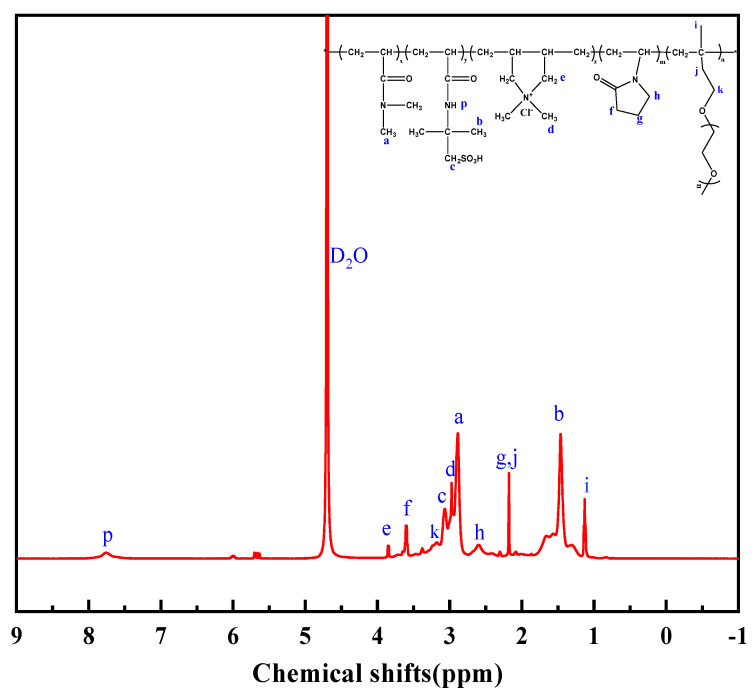
^1^H NMR spectrum of DNDAP.

The results of the NMR spectroscopy with the peak assignments are shown in Figure 2. The chemical shift peaks of –CH_3_ in AMPS and CH_3_ in DMAA are 1.46 and 2.88, respectively. The chemical shift peaks of N-CH_2_ in NVP, O-CH_2_ in TPEG, as well as -CH_3_ and -CH_2_-CH- in DMDAAC are 2.62, 3.17, 2.97, and 3.85 ppm, respectively. Based on Figure 1 and Figure 2, it can be concluded that the synthesized DNDAP possesses the characteristic functional groups and chemical shift peaks of all monomers, suggesting that it is the intended product.

**Figure 3:**
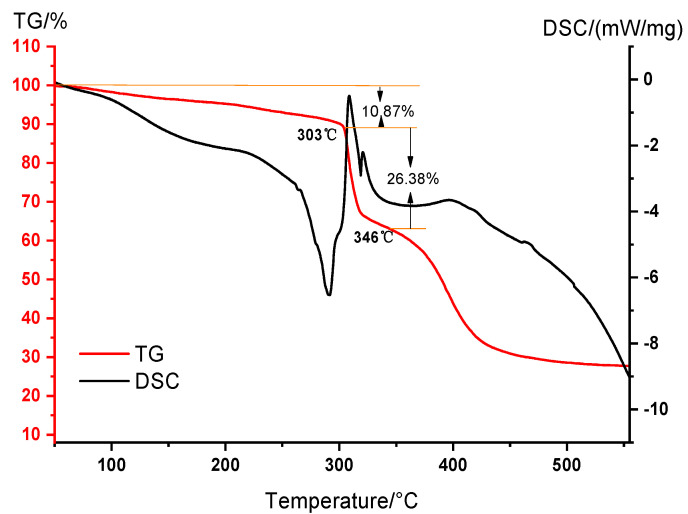
TGA-DSC analysis of DNDAP.

The thermogravimetric analysis (TGA) method was used to determine the thermal stability of the DNDAP copolymer. The results of the TGA experiments are shown in Figure 3. Based on the figure, weightlessness can be divided into three stages from room temperature to 610 °C. In the first stage, the temperature ranges from room temperature to 303 °C. As the temperature rises, the mass decreases slowly, and the TGA curve is flat at this point. It is estimated that 10.87% of the mass has been lost during this stage. Due to the existence of a large number of amide, sulfonic acid, and quaternary ammonium groups in the DNDAP copolymer, which are strong polar hydrophilic groups, the copolymer easily adsorbs water molecules in the air or interacts with water molecules. The main cause of mass loss is the gradual evaporation of water and volatile components in the DNDAP polymer with the increase in temperature. The temperature of the second stage is 303–346 °C, and the weight loss rate of this stage is 26.38%. The weight loss rate is higher relative to the first stage. It is caused by the decomposition and volatilization of the amide group in the copolymer and the absorption of a large amount of heat. A third stage of the process occurs between 346 and 610 °C. During this stage, the C-C bond in the copolymer backbone breaks as the temperature rises. The results show that the initial decomposition temperature of the DNDAP copolymer is 303 °C. Before that, the functional groups in the copolymer are stable, and the polymer has good thermal stability.

### 2.2. Filtration Loss Reduction Performance of DNDAP in the WBDF

**Figure 4:**
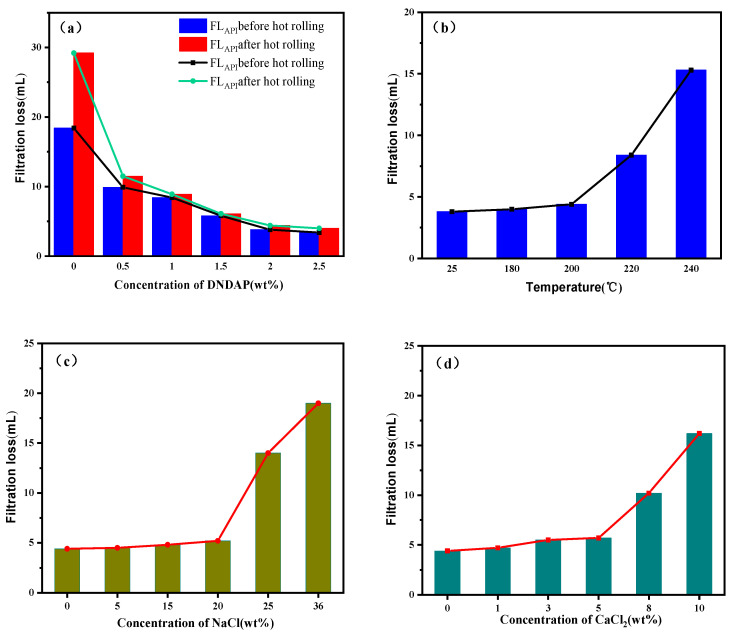
Filtration performance of DNDAP in the WBDF. (**a**) WBDF containing various concentrations of DNDAP and its filtration loss before and after aging at 200 °C for 16 h. (**b**) Filtration performance of the WBDF supplemented with 2 wt% DNDAP at various temperatures. (**c**) The filter loss of DNDAP in Na-WBDF at different concentrations after aging at 200 °C for 16 h. (**d**) The filter loss of DNDAP in Ca-WBDF at different concentrations after aging at 200 °C for 16 h.

As a result of the pressure difference, the free water in the drilling fluid penetrates into the cracks or pores of the rock on the borehole wall, and the solid particles in the drilling fluid attach to the borehole wall and form mud cake, thus preventing or slowing the further invasion of the drilling fluid into the formation. During drilling, drilling fluid filtration mainly refers to the filtration loss and the quality of the mud cake formed, which plays a crucial role in ensuring wellbore stability. Drilling fluids are commonly evaluated based on American Petroleum Institute (API) standards. It can be seen from Figure 4a that the filtration loss increases significantly after drilling fluid aging, indicating that high temperature affects the performance of bentonite, and the filtration control ability of filter cake decreases. In the case of both drilling fluids before and after aging, filtration loss decreases gradually with increasing DNDAP concentration. Adding 0.5 wt% DNDAP significantly reduced the drilling fluid filtration loss from 18.4 mL to 9.9 mL before aging and 29.2 mL to 11.5 mL after aging. When the DNDAP addition was increased to 2 wt%, the filtration loss was reduced from 18.4 mL to 3.8 mL before aging and from 29.2 mL to 4.4 mL after aging. As DNDAP dosage is continuously increased, the fluid loss reduction volume of the drilling fluid continues to remain relatively unchanged, and the performance of fluid loss reduction does not improve significantly. Accordingly, 2 wt% DNDAP is recommended from the standpoint of cost. Figure 4b shows the filtration loss of DNDAP/WBDF with a DNDAP content of 2 wt% after aging at different temperatures. As a result of aging the drilling fluid at 200 °C for 16 h, the filtration loss decreased from 29.2 mL to 4.4 mL. Upon aging at 220 °C for 16 h, the filtration loss decreased to 8.4 mL, indicating that DNDAP exhibited good anti-high-temperature filtration loss properties. When different concentrations of NaCl were added to DNDAP/WBDF, the filtration loss of the drilling fluid increased, as shown in Figure 4c. The filtration loss of the drilling fluid is 5.2 mL when the NaCl dosage is 20 wt%. Upon adding NaCl at 25 wt%, the filtration loss increased sharply to 14.0 mL. Compared to WBDF without DNDAP, the filtration loss after aging decreased from 138.2 mL (Table 1) to 5.2 mL after contamination with 20 wt% NaCl. As a result, DNDAP is resistant to NaCl pollution after aging at high temperatures. When different concentrations of CaCl_2_ were added to DNDAP/WBDF, the filtration loss of the drilling fluid increased gradually, as shown in Figure 4d. The fluid loss is 5.7 mL when CaCl_2_ is added at 5 wt%. Adding CaCl_2_ at 8 wt% results in a fluid loss of 10.2 mL. Compared to WBDF without DNDAP, the filtration loss after aging decreased from 143.8 mL (Table 1) to 5.7 mL after contamination with 5 wt% CaCl_2_. This indicates that DNDAP still has good anti-CaCl_2_ pollution ability after high-temperature aging. The results show that, under the same conditions, the influence of divalent calcium ions on the fluid loss reduction performance of the WBDF is greater than that of monovalent sodium ions. In addition, DNDAP exhibits superior filtration performance in saline-calcium water-based drilling fluids.

### 2.3. Rheological Properties of DNDAP-Based Drilling Fluid

**Figure 5:**
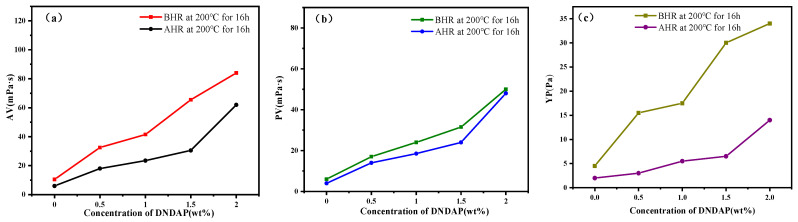
Rheological parameters of different concentrations of DNDAP before (BHR) and after (AHR) aging in the WBDF (**a**–**c**).

It is critical that drilling fluids have adequate rheological properties to carry and suspend cuttings as well as to maintain wellbore stability. Therefore, the viscosity characteristics of DNDAP in water-based drilling fluids were examined. As shown in Figure 5, the rheological parameters of the base drilling fluid were low but increased with the addition of the DNDAP polymer. With the increase in DNDAP dosage to 2 wt%, AV increased from 9 mPa·s to 80 mPa·s, PV increased from 5 mPa·S to 45 mPa·s, and YP increased from 4.5 mPa·s to 32 mPa·s. It is evident from the results that the copolymer possesses excellent rheological properties. In general, when the AV value in the WBDF exceeds 80 mPa·s, the fluidity of the WBDF is weakened, and drilling accidents can occur, such as sticking. Based on the filtration reduction performance of DNDAP, as well as its economy and safety, 2 wt% is the recommended dosage of DNDAP. The rheological properties of DNDAP in water-based drilling fluid are slightly reduced after aging at high temperatures, but the changes are not significant, and the rheological characteristics continue to meet drilling demands after aging, which is better than the rheological properties of basic drilling fluids.

**Figure 6:**
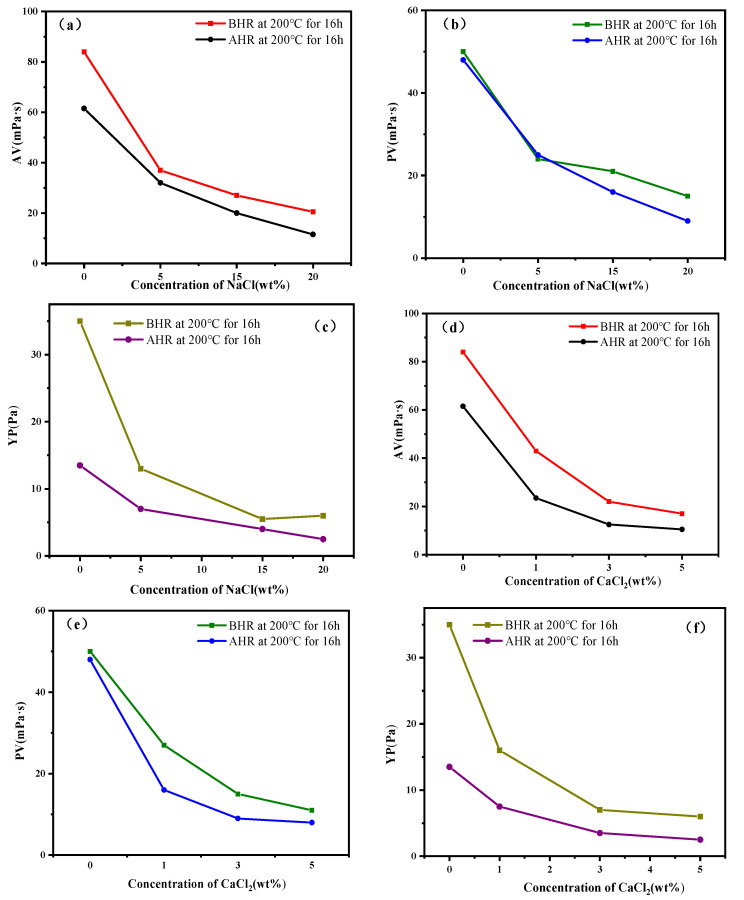
Rheological parameters of DNDAP before and after aging in WBDF with different salt and calcium concentrations (**a**–**f**).

The filtration loss reduction effect of DNDAP was still effective after high-temperature aging when it was added to WBDF contaminated with 20 wt% NaCl or 5 wt% CaCl_2_. A series of experiments was conducted to evaluate whether the rheological properties of DNDAP in WBDF contaminated with calcium salts meet the requirements. As shown in Figure 6, with the increase in NaCl or CaCl_2_ content, the viscosity of the WBDF decreased continuously. When the NaCl content increased to 20 wt%, the AV of DNDAP/WBDF dropped from 62 to 14 mPa·s, the PV decreased from 50 to 9 mPa·s after high-temperature aging, and the YP decreased from 12 to 4 Pa. The AV of DNDAP/WBDF decreased from 62 mPa·s to 12 m·s, the PV fell from 50 mPa·s to 9 mPa·s, and the YP decreased from 12 Pa to 4 Pa after aging at 200 °C for 16 h with the increase in CaCl_2_ content to 5 wt%. The rheological parameters of DNDAP/WBDF decreased more after salt and calcium contamination because the DNDAP polymer chain was cowering after adding cations, resulting in a decrease in viscosity. Nevertheless, it is sufficient to meet drilling demands.

### 2.4. Mechanism Analysis

#### 2.4.1. Morphological and Elemental Analysis of Mud Cake Surface

**Figure 7:**
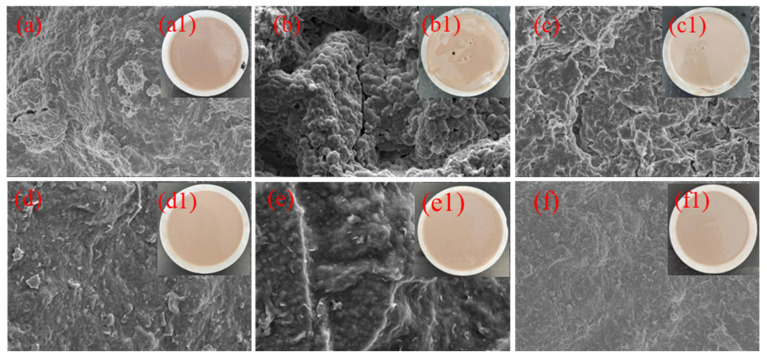
(**a**−**f**) SEM of filter cakes; (**a1**−**f1**) digital images of filter cakes; (**a**,**a1**) WBDF; (**b**,**b1**) 20Na -WBDF; (**c**,**c1**) 5Ca-WBDF; (**d**,**d1**) DNDAP/WBDF; (**e**,**e1**) DNDAP/20Na -WBDF; (**f**,**f1**) DNDAP/5Ca-WBDF.

A filter cake’s density can be used to determine the filtration performance of drilling fluids. Typically, thin and dense filter cakes have low permeability and small fluid losses. Conversely, a thick, loose filter cake may have higher permeability and porosity, thus resulting in greater fluid loss. As shown in Figure 7, digital images and SEM images were obtained after the aging of the filter cake. Figure 7 shows filter cakes’ digital images and SEM images after drilling fluid aging. As seen from Figure 7(a1), after high-temperature aging of water-based drilling fluid, the filter cake is thicker but the surface is smooth. The results of further analysis by SEM revealed that bentonite accumulated on the surface of the filter cake, with pores and cracks clearly visible in Figure 7a. Based on this comparison, it can be concluded that high temperatures change the characteristics of filter cakes. As shown in Figure 7(b1,c1), when NaCl or CaCl_2_ are added to the water-based drilling fluid, the thickness of the filter cake increases and small holes appear on the surface. In this case, the filter cake’s ability to block water is significantly reduced. The fluid can easily penetrate the filter cake, resulting in a large loss of drilling fluid. Furthermore, the macroscopically large amount of filtration loss is consistent with this conclusion. Because of the high temperature and the cations (Na^+^ and Ca^2+^), the flocculation and aggregation of bentonite particles in the drilling fluid are aggravated, resulting in increased porosity of the filter cake and an inability to plug the filtrate. Upon aging at high temperatures, the filter cakes obtained after adding 2 wt% DNDAP to the WBDF, 20 Na-WBDF, and 5 Ca-WBDF were significantly thinner than the filter cakes without DNDAP. As seen in Figure 7(e1,f1), the drilling fluid containing DNDAP formed thin and dense filter cakes without pores on their surface. Scanning electron microscopy demonstrated that almost no bentonite particles accumulated on the filter cake surface and that no pores or cracks developed, as shown in Figure 7d–f. DNDAP has proven effective due to improved filter cake densification and low permeability. The addition of DNDAP can hinder the synergistic destruction caused by high temperatures and high salt concentrations, indicating that DNDAP is highly resistant to high temperatures and high salt concentrations.

**Figure 8:**
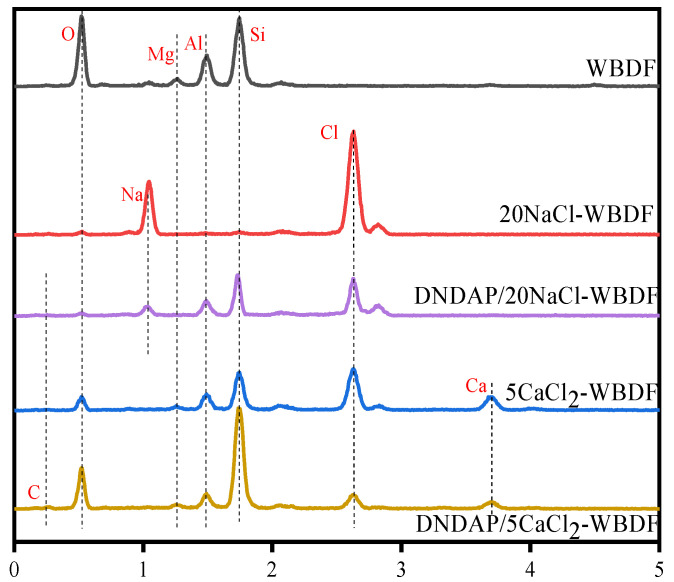
EDS element analysis of filter cakes.

EDS is used to explain the role of DNDAP, as shown in Figure 8. For the WBDF, peaks were observed for aluminosilicate, including the elements silicon, aluminum, magnesium, and oxygen. Nevertheless, when the WBDF was contaminated with 20 wt% NaCl or 5 wt% CaCl_2_, aluminosilicate peaks were weak or even disappeared, while Na, Ca, and Cl peaks appeared. This means that the surface of the bentonite particles was covered with an abundance of inorganic salts. When DNDAP was added to salt-contaminated WBDF, the peak caused by inorganic salt decreased sharply, while the peak caused by aluminosilicate recovered. In the filter cake, the content of sodium and chlorine was significantly reduced, which may be due to the fact that part of the DNDAP copolymer was well adsorbed on the bentonite particles during the formation of the mud cake, preventing cations from entering the bentonite. A complex is formed when the cation penetrates the ion network structure of the DNDAP copolymer.

#### 2.4.2. Particle Size Distribution Test

**Figure 9:**
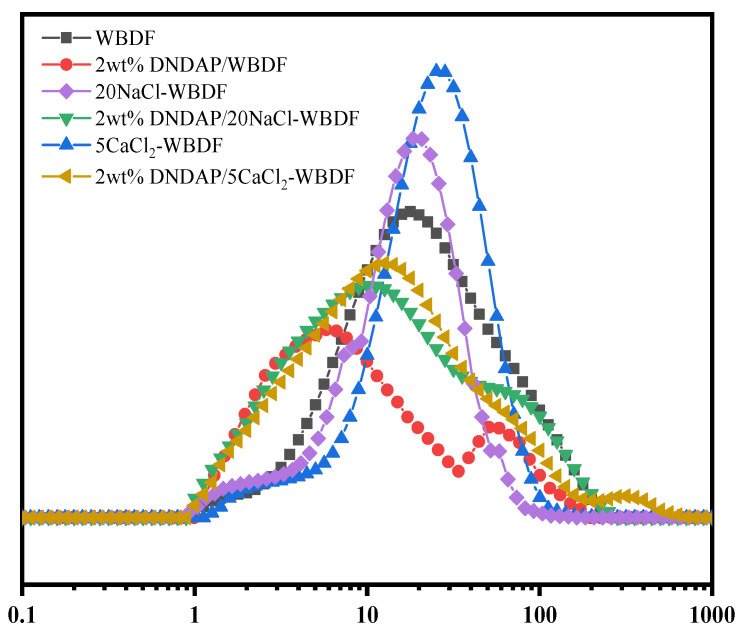
Size distribution of the WBDF under different conditions after aging at 200 °C for 16 h.

As a result of pressure differences, solid particles in drilling fluids adhere to the surface of the well wall to form a mud cake. A thin and dense mud cake is necessary to reduce the filtration loss of the drilling fluid, so the bentonite in the water-based drilling fluid must contain a combination of large and small particles. The large particles are used as bridging particles, while the small particles are used as fillers. Therefore, the particle size distribution of the drilling fluid system should be reasonable. Figure 9 shows the particle size distribution of each system after aging. The particle size distribution curve shifted to the left after adding 2 wt% DNDAP to the WBDF, changing from unimodal to bimodal. The results show that DNDAP can be adsorbed on the bentonite particles and form a polymer adsorption layer after high-temperature aging, thus reducing the probability of collision and coalescence between particles. The bentonite particles maintain good dispersion, have a reasonable particle size distribution, make the formation of mud cake dense, and finally achieve the effect of reducing filtration loss. The particle size distribution curve of the WBDF shifted to the right when 20 wt% NaCl or 5 wt% CaCl_2_ were added. Consequently, high salt levels will result in flocculation and aggregation of bentonite particles, increasing size and destroying their distribution range. The particle size distribution curves were shifted to the left after 2 wt% DNDAP was added to 20NaCl-WBDF or 5CaCl_2_-WBDF, and the range of particle sizes became wider as a result. The results show that DNDAP adsorbed on bentonite particles under high salt conditions can reduce the effect of Na^+^ or Ca^2+^ on bentonite particles and promote the dispersion of bentonite particles. The system has a wider particle size distribution and more reasonable particle size grading, which is conducive to forming dense mud cakes. As a consequence of the results, it appears that DNDAP can optimize the WBDF’s size distribution under conditions of high temperature or high calcium salt concentration to ensure its stability under such conditions.

#### 2.4.3. Zeta Potential Test

**Figure 10:**
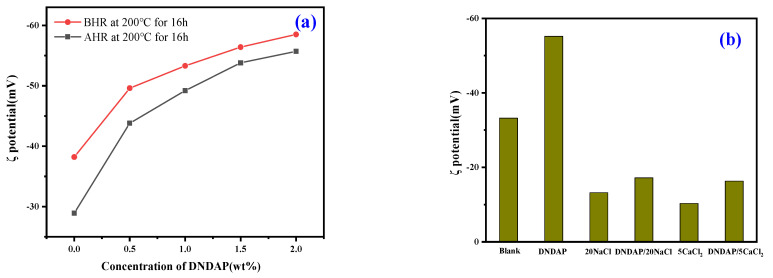
Zeta potential value of WBDF.

WBDF is a colloidal dispersion system composed of bentonite and water. Zeta potential is an effective indicator used to evaluate the dispersion stability of colloids. According to Figure 10a, as the concentration of DNDAP increases, the absolute value of the drilling fluid zeta potential gradually increases. The results show that the copolymer has a strong adsorption effect on bentonite particles, thus enhancing its dispersion stability. Zeta potentials of drilling fluids have been observed to decrease after high-temperature aging. As a result of the increased thermal motion of water molecules caused by the high temperature and the thinned hydration film layer on bentonite particles, the dispersion stability is weakened. As can be seen from Figure 10b, the absolute value of the zeta potential decreased significantly after Na^+^ and Ca^2+^ were added to the solution. As the concentration of cations increases, the absolute value of the zeta potential gradually decreases. As a result, the zeta potential for 20Na-WBDF and 5Ca-WBDF decreased to −13.2 and −10.3 mV, respectively. The results show that the addition of inorganic Na^+^ and Ca^2+^ inhibited the double electric layer and increased colloidal instability, resulting in increased bentonite particle aggregation and flocculation. The absolute value of the zeta potential increased to −55.2 mV when 2 wt% DNDAP was added to the WBDF, indicating that DNDAP improved the stability of the solution. Upon addition of DNDAP to 20Na-WBDF and 5Ca-WBDF, zeta potentials recovered to −17.2 mV and −16.3 mV, respectively. As can be seen, the addition of DNDAP is still beneficial to the colloid’s stability under conditions of high salt concentration. In this case, the positively charged portion of the DNDAP polymer attaches to bentonite particles, while the negatively charged portion acts as a barrier, thereby improving the stability of the dispersion. When contaminated by cations, DNDAP adsorbs cations, minimizing the adsorption of cations by bentonite particles and preventing further coalescence of bentonite colloids, thus maintaining their dispersion stability.

## 3. Conclusions

An amphoteric copolymer gel, DNDAP, was prepared by free radical polymerization of DMAA, NVP, DMAAC, AMPS, and TPEG as a novel high-temperature-resistant and calcium salt contamination-reducing filter loss agent. The thermographic analysis showed that DNDAP exhibited high thermal stability. In addition, the addition of 2.0 wt% DNDAP to the WBDF can effectively control fluid loss at 200 °C with 20 wt% NaCl or 5 wt% CaCl_2_ contamination and has excellent filtration loss reduction performance. The denseness of the filter cake was significantly improved under both freshwater and brine conditions with the addition of DNDAP. At the same time, the addition of DNDAP improved the colloid stability of the calcium salt-contaminated WBDF. It prevented bentonite particles from coalescencing under high temperatures, salinity, and calcium. The mechanism analysis shows that DNDAP produces strong adsorption of bentonite particles in the WBDF through sulfonic acid groups and quaternary ammonium salts. It maintains a reasonable particle size distribution of bentonite particles and forms a dense mud cake, thus achieving the effect of reducing filtration loss. This amphoteric polymer material shows good prospects for application, especially in oil and gas extraction in deep wells and even ultra-deep wells drilled under extremely harsh conditions.

## 4. Materials and Methods

### 4.1. Materials

N, N-dimethylacrylamide (98%), N-vinylpyrrolidone (99%), dimethyl diallyl ammonium chloride (60%), and 2-acrylamide-2-methylpropanesulfonic acid (98%) were purchased from Beijing Bailingway Technology Co., Ltd. (Beijing, China). Isopentenol polyether (TPEG, 99%) was purchased from Wuhan lullaby pharmaceutical chemical Co., Ltd. (Wuhan, China). The remaining reagents were analytical grade and purchased from Shanghai Aladdin Biochemical Technology Co., Ltd. (Shanghai, China). without further purification.

### 4.2. Synthesis of Copolymer DNDAP

**Figure 11:**
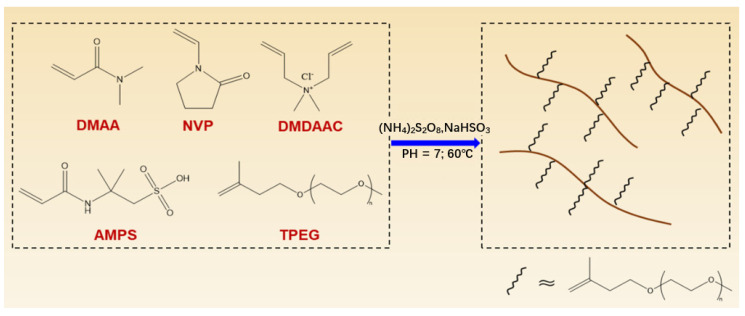
The synthesis procedure of the DNDAP copolymer.

The copolymer DNDAP was synthesized by free radical polymerization. The monomer was dissolved in deionized water according to a molar mass ratio of *n* _DMAA_: *n* _NVP_: *n* _DMDAAC_: *n* _AMPS_ = 5:3:2:2; the pH of the monomer solution was adjusted to 7 by adding NaOH solution, and then 1% (molar ratio) of the total TPEG monomer was added. Under nitrogen protection, the above solution was poured into a four-necked flask and uniformly mixed for 30 min. For further initiation, 0.5 wt% ammonium persulfate and sodium bisulfite were added as initiators to react at 55 °C for 4 h. In the subsequent step, the product was washed, precipitated, and filtered using anhydrous ethanol–acetone. Finally, the DNDAP gel was obtained. A diagram of the synthesis process is shown in Figure 11.

### 4.3. Characterization of DNDAP

Fourier transform infrared spectroscopy (Nicolet Is10 FTIR spectrometer, Nicolet, Wisconsin, USA) was used to characterize the DNDAP copolymer. Infrared absorption spectra between 4000 and 400 cm^−1^ were measured for the purified DNDAP copolymer powder by KBr tableting. The ^1^H NMR spectrum of the DNDAP polymer was measured using a Bruker NMR spectrometer (Bruker AVANCE III 600 M nuclear magnetic resonance apparatus, Bruker, Germany). The mass change of the DNDAP copolymer that occurred from 40 °C to 610 °C at a heating rate of 4 °C per min under N_2_ protection was measured using a thermal analyzer (STA449 F5 synchronous thermal analyzer, Netzsch, Germany).

### 4.4. Preparation of the WBDF

Add 40 g of bentonite and 2 g of Na_2_CO_3_ to 1000 mL of deionized water, stir at high speed for 20 min, scrape off the bentonite on the cup wall twice in the middle, and then stabilize under sealed conditions for 24 h. The water-based drilling fluid (WBDF) is successfully prepared. Several experiments were conducted to evaluate the filtration performance and salt tolerance of DNDAP in the WBDF. In this paper, WBDF supplemented with NaCl or CaCl_2_ was labeled as *X* Na-WBDF or *X* Ca-WBDF, respectively. WBDF with DNDAP added was labeled as DNDAP/WBDF. The *X* Na-WBDF or *X* Ca-WBDF supplemented with DNDAP were labeled as DNDAP/*X* Na-WBDF or DNDAP/*X* Ca-WBDF, respectively. *X* is the mass percentage of NaCl or CaCl_2_ added to the solution.

### 4.5. Filtration Performance and Rheological Properties of DNDAP/WBDF

In accordance with API guidelines, the filtration capacities of freshly prepared drilling fluid and drilling fluid aged for 16 h were evaluated. Approximately 250 mL of drilling fluid was passed through an SD-3 medium-pressure filter device (Qingdao Haitongda Special Instrument Co., LTD., Qingdao, China) for 30 min at a pressure difference of 0.69 MPa.

A ZNN-D6 six-speed viscometer (Qingdao Haitongda Special Instruments Co., Ltd., Qingdao, China) was used to study the rheological characteristics of DNDAP/WBDF at different rotational speeds. The rotational speeds were recorded as θ_600_, θ_300_, θ_200_, θ_100_, θ_6_, and θ_3_ in descending order. Based on Equations (1) to (3) below, we calculated the apparent viscosity (AV), plastic viscosity (PV), and yield point (YP) of DNDAP/WBDF.

Apparent viscosity (AV) = 0.5θ_600_ (mPa·s) (1)

Plastic viscosity (PV) = θ_600_ − θ_300_ (mPa·s) (2)

Yield point (YP) = 0.511 × (θ_300_ − PV) (Pa) (3)

### 4.6. Mechanism Analysis

A scanning electron microscope (SEM) (Quanta FEG250, Hillsboro, USA) was used to examine the microscopic morphology and elemental analysis of filter cake surfaces. The cake obtained from the filtration loss experiment was adhered to the copper plate, sprayed with gold for 10 min, and then tested.

For the study of WBDF stability, a zeta potential analyzer (Malvern Zetasizer Nano Z, Nottingham, UK) was employed. After a tenfold dilution of the WBDF, its zeta potential was measured. To ensure the accuracy of the test, each group of experiments was repeated three times, and the average value obtained was recorded as the zeta potential value

A Mastersizer 2000 laser particle size instrument (Malvern, UK) was used to examine the particle size distribution of the particles.

## Figures and Tables

**Table 1:** The Fluid Loss of the WBDF at Different Temperatures and Salt Concentrations.

C (Bentonite) (wt%)	C (Na^+^) (wt%)	C (Ca^2+^) (wt%)	T (℃)	FL_API_ (mL)
4	0	0	25	18.4
			200	29.2
	20	0	25	84.6
			200	138.2
	0	5	25	57.6
			200	143.8

Note: C (bentonite): Amount of bentonite, C (Na^+^) and C (Ca^2+^): concentration of NaCl and CaCl_2_, FL_API_: API fluid loss.

## Data Availability

Not applicable.
